# The Exploration of miRNAs From Porcine Fallopian Tube Stem Cells on Porcine Oocytes

**DOI:** 10.3389/fvets.2022.869217

**Published:** 2022-05-09

**Authors:** Tzu-Yen Fu, Shu-Hsuan Wang, Tzu-Yi Lin, Perng-Chih Shen, Shen-Chang Chang, Yu-Han Lin, Chih-Jen Chou, Yu-Hsiang Yu, Kuo-Tai Yang, Chao-Wei Huang, Steven W. Shaw, Shao-Yu Peng

**Affiliations:** ^1^Department of Animal Science, National Pingtung University of Science and Technology, Pingtung, Taiwan; ^2^College of Medicine, Chang Gung University, Taoyuan, Taiwan; ^3^Kaohsiung Animal Propagation Station, Livestock Research Institute, Council of Agriculture, Executive Yuan, Pingtung, Taiwan; ^4^Institute of Biotechnology, National Taiwan University, Taipei, Taiwan; ^5^Department of Biotechnology and Animal Science, National Ilan University, Yilan, Taiwan; ^6^Department of Tropical Agriculture and International Cooperation, National Pingtung University of Science and Technology, Pingtung, Taiwan; ^7^Department of Obstetrics and Gynecology, Taipei Chang Gung Memorial Hospital, Taipei, Taiwan; ^8^Prenatal Cell and Gene Therapy Group, Institute for Women's Health, University College London, London, United Kingdom

**Keywords:** extracellular vesicles, fallopian tube, microRNA, miR-320a-3p, oocytes

## Abstract

Fallopian tube is essential to fertilization and embryonic development. Extracellular vesicles (EVs) from Fallopian tube containing biological regulatory factors, such as lipids, proteins and microRNAs (miRNAs) serve as the key role. At present, studies on oocytes from porcine oviduct and components from EVs remain limited. We aim to explore the effect of EVs secreted by porcine fallopian tube stem cells (PFTSCs) on oocyte. When the fifth-generation PFTSCs reached 80–90% of confluency, the pig *in vitro* maturation medium was utilized, and the conditioned medium collected for oocyte incubations. To realize the functions of EVs, several proteins were used to determine whether extracted EVs were cell-free. Field emission scanning electron microscope and nanoparticle tracking analyzer were used to observe the morphology. By next generation sequencing, 267 miRNAs were identified, and those with higher expression were selected to analyze the Gene Ontology and Kyoto Encyclopedia of Genes and Genomes pathway enrichment maps. The selected miR-152-3p, miR-148a-3p, miR-320a-3p, let-7f-5p, and miR-22-3p, were predicted to target Cepb1 gene affecting MAPK pathway. Of the five miRNAs, miR-320a-3p showed significant difference in maturation rate *in vitro* maturation. The blastocyst rate of pig embryos was also significantly enhanced by adding 50 nM miR-320a-3p. *In vitro* culture with miR-320a-3p, the blastocyst rate was significantly higher, but the cleavage rate and cell numbers were not. The CM of PFTSCs effectively improves porcine oocyte development. The miRNAs in EVs are sequenced and identified. miR-320a-3p not only helps the maturation, but also increases the blastocyst rates.

## Introduction

With the change of life style and advancement of maternal age, premature ovarian failure, endometriosis, myoma, and endometrial polyp become highly associated with infertility (1). The aging and damage of cells would produce excessive reactive oxygen species (ROS) and decrease the ability of anti-oxidation (2, 3). The imbalance of reduction-oxidation reaction leads to the deposition of ROS affecting the function of chromosome and mitochondria (4, 5). Also, these abnormalities retard the development of oocytes and ovaries (6).

Extracellular vesicles (EVs) from the fallopian tube play an important role in intercellular communication (7). This carrier of bioactive material is essential to the physiology of gamete, the success of fertilization, and the early growth of embryo. EVs from bovine fallopian tube fluid are able to improve the activity of sperm and elevate the concentration of calcium for capacitation. Besides, bovine EVs from fallopian tube epithelium could increase blastocyst rate *in vitro* culture (8).

EVs contain several bioactive materials, such as lipid, RNA, mRNA, and microRNA (miRNA). Genetic modification could be conducted with the expression of miRNAs in target cells. For instance, miR-30b, let-7, miR-181a, miR-375, miR-503, and miR-513a-3p from follicular fluid-derived EVs have the capability of modulating the development of follicles and the maturation of oocytes (9–13).

To sum up, EVs from the fallopian tube fluid seems to ameliorate damages from aging and environment factors. Additionally, miRNAs of EVs are able to combine with the gene of target cells and improve the growth of oocytes and embryos. Nowadays, studies in both the association between fallopian tube and oocyte and the type of miRNAs of EVs from the fallopian tube remain limited. To improve the *in vitro* culture system to assist in the development of ovum, porcine fallopian tube stem cell (PFTSC) was utilized to investigate the influence of EVs on the oocyte. Meanwhile, we aim to analyze the miRNAs expression profiles of EVs from PFTSC so as to identify the key factor affecting the growth of oocyte.

## Materials and Methods

### Animal Source

Pig (Landrace x Yorkshire x Duroc) fallopian tubes were collected and washed with warm saline containing 1% penicillin/streptomycin (P/S, GibcoTM, 15140122, E.U. Approved, South American) in the abattoir in Pingtung County, so the approval from institutional animal care and use committee were waived. The tissue was taken to National Pingtung University of Science and Technology (NPUST) in 2 h.

### Isolation of Porcine Fallopian Tube Stem Cells

After three times wash with saline containing 1% P/S, porcine fallopian tubes were minced into 0.1–0.5 mm3. The tissue pieces were incubated with cell culture media (Alpha MEM, Sigma, M0894, 3050 Spruce Street, SL Louis, MO, USA) supplemented with 10% fetal bovine serum (FBS, GibcoTM, 10270106, E.U. Approved, South America) and 1% P/S in a 10 cm culture dish at 38.5°C and 5% CO_2_. The isolation was successfully conducted by our previously published approach. The expressions of CD44-FITC, CD105-PE, and CD4-PE in porcine fallopian tube stem cells of this study were 76.7, 7.6, and 3.7%, respectively. That is, these cells shared similar potential with stem cells and were able to serve as good subject for this experiment (14).

### Porcine Follicular Fluid Preparation

Porcine ovaries obtained from the abattoir were kept in warm saline containing 0.1 mg/ml penicillin-streptomycin (P/S; Gibco, 15140-122, USA) and brought back to NPUST in 2 h. After several times of wash, porcine follicular fluid (PFF) was collected from follicles with a diameter of 3–6 mm. Fluid was centrifuged at 3,300 x g for 10 min, then collected the supernatant and filtered (0.45 and 0.22 μm in sequence). Store at−20°C for use.

### PFTSC-Conditioned Medium Production

The conditioned medium was prepared following the previous report. Once PFTSC on the 5th passage reached 80–90% confluence in a 10 cm culture dish, the culture medium was removed, and cells were washed with DPBS. Then, cells were incubated in 3 ml tissue culture medium 199 (TCM199) containing M199 (medium 199, Sigma, m4530) supplemented with 10% Defined Fetal Bovine Serum (Defined FBS; HyClone, SH30070.03, USA), 2.5 μg/ml follicle-stimulating hormone (FSH, Sigma, F-2293), 5 IU/μl human chorionic gonadotropin (hCG, Biovision, 4778-1000 155, S. Milpitas Boulevard, Milpitas, CA 95035, USA), 10 ng/ml EGF(Sigma, E-4127), 1% antibiotic-antimycotic (ABAM, Gibco,15240-062, Grand Island, New York, USA), and 10% PFF (v/v) for 3 h at 38.5°C and 5% CO2. The PFTSC- conditioned medium was collected and filtered (0.22 μm) and stored at 4°C for use.

### Isolation of EVs From PFTCs

For isolation of EVs, general FBS was replaced to 10% (v/v) exosome-depleted FBS (Gibco, A27208-01) in PFTSC-conditioned medium. PFTCs-conditioned medium was mixed with miRCURY Exosome Kit (Qiagen, 76743, BRD), and incubated at 4°C for 1 h. Then, centrifuge at 3,200 x g for 30 minutes. The supernatant was discarded, and the pellet was stored in the resuspension buffer at −20°C.

### Electron Microscopy

After thawing the stored solution containing EVs, the supernatant was removed and the exosome pellet was re-suspended with an appropriate volume of glutaraldehyde. 10 μl solution was added onto a glass slide and dried. Samples were coated with gold-palladium alloy, and observed by a scanning electron microscope (SEM, HITACHI, S3000N, JPN) at a magnification of 20,000X. For TEM, the exosome pellet was re-suspended by cold DPBS. Exosomes were mounted on copper grids, fixed by glutaraldehyde in cold DPBS for 5 min. After wash by sterile distilled water, contrasted by uranyl-oxalate solution at pH 7 for 5 min, excessive fluid was removed and dried. (Transmission Electron Microscope, TEM; HITACHI, 7500, JPN) A HITACHI 7500 TEM was used to image exosome at a magnification of 100,000X. Imaging was performed in Precision Instruments Center of NPUST.

### Nanoparticle Tracking Analysis

The data of exosome size distribution and concentration were collected using a NanoSight LM10HS (Malvern Panalytical, UK) in the Center for Micro/Nano Science and Technology of National Cheng Kung University. Particles were tracked and sized automatically with Nanoparticle tracking analysis (NTA). The background was measured by filtered PBS.

### Western Blot

Cell lysates and exosome pellets were subjected to 12% sodium dodecyl sulfate–polyacrylamide gel electrophoresis (SDS-PAGE). 40 μg of total protein were denatured in electrophoresis sample buffer and boiled at 95°C for 5 min before running SDS-PAGE. Afterward, proteins were transferred into polyvinylidene fluoride (PVDF) membranes. After blocking, the PVDF membranes were probed with antibodies for actin (1:5,000, Thermo Fisher, MA5-11869, USA), cytochrome c (1:5,000, Proteintech, 10993-1-AP, USA), HSP70 (1:5000, Bio-rad, MCA6096, USA) at 4°C for 12 h. After several thorough washes with Tween-Trisbuffered saline (TTBS), the PVDF membranes were incubated with the secondary antibodies in TTBS at room temperature for 1 h. After washing with TTBS, the PVDF membranes were visualized using Immobilon Crescendo Western horseradish peroxidase (HRP) substrate (Merck, WBLUR0500), according to the manufacturer's instructions.

### MicroRNA Extraction

After obtained exosome pellets, exosomal microRNAs (exomiRs) were isolated using a miRCURY Exosome Cell/Urine/CSF Kit (QIAGEN, Cat No.76743) following the manufacturer's procedure. The microRNA concentration was quantified by Qubit microRNA Assay Kit on Qubit Fluorometer (Thermo Fisher, USA) and used microfluidic electrophoresis to qualify the microRNA length distribution ~22 nts with RNA Kit on MultiNA MCE-202 (Shimadzu, Japan).

### Next Generation Sequencing

A total of 10 ng exomiRs were used for library preparation, following the manual of VAHTS Small RNA Index Primer Kit (Vazyme, NR801, China). The correct barcoding adapter-ligated library amplicon length will be around 142 bp and amplicons were purified using FastPure Gel DNA Extraction Mini Kit (Vazyme Biotech, CN). The concentration was quantified using microfluidic electrophoresis and the length of the eluted barcoding adaptor-ligated library was checked by DNA-2500 Kit on MultiNA MCE-202 (Shimadzu, Japan). The qualified pooled library (4nM) was sequenced on an Illumina NextSeq 500 (single-end, 75 sequencing cycles) (Illumina, USA). NGS was performed twice, and 14 most abundance of miRNAs in conditioned PFTSC (**Table 2**) were selected to further analysis and experiments.

### Bioinformatic Analysis and Target Genes Prediction

Firstly, total reads were trimmed 34bp on 3' end to remove the adapter sequence by Clip (Galaxy Version 1.0.3). Then correct insert size around 15-30bp for mature exomiRs was retained by Filter FASTQ (usegalaxy.org). The trimmed reads were mapped with pig microRNA database v22.1 (miRbase.org) using BWA (Galaxy Version 0.7.17.4). Then, the mapped reads of each sample were assembled and quantified using Linux command. The target genes of highly differentially expressed exomiRs were predicted from Target scan v7.2 (targetscan.org), miRanda (www.microrna.org) and miRDB (http://www.mirdb.org/). The union of target genes was conducted by the Gene ontology (GO) and Kyoto Encyclopedia of Genes and Genomes (KEGG) pathway enrichment analysis by ClusterProfiler (R package) *p* value < 0.05, *q* value < 0.05), and using ggplot2 (R package) to visualize the enrichment plots.

### Preparation of MiRNAs

A total of 5 kinds of Cpeb1-related 2'-O-methyl modified miRNAs were synthesized. Each miRNA was prepared in RNase-free water to a 20 μM stock solution at−20°C and capsulated using a liposome-based HiPerFect Transfection Reagent (301707, QIAGEN). Before the cultivation of oocyte, 1 μl of 5 Cpeb1-related 2'-O-methyl modified miRNAs were added to the mixture of 3 μl and stored at room temperature for 5–10 min. Then, TCM199 culture medium was added to achieve the miRNA concentration of 200nM. After the maturation of oocytes, certain miRNA would be added.

### Porcine Oocytes *in vitro* Maturation (IVM)

The porcine ovaries were gained from the abattoir and taken to NPUST with warm- saline containing 1% P/S in 1 h. After wash, the follicle fluid was aspirated from ovaries and collected in 15 mL centrifuge tubes. Keep warm at 37°C in a water bath for 5 min to precipitate oocytes. Cumulus-oocyte complexes (COCs) were collected by a hand-made glass needle from the precipitated fluid. For IVM, COCs were washed twice by DPBS containing 1% bovine serum albumin and transferred to maturation medium or PFTSC conditioned medium, which is covered by paraffin liquid (NacalaiTesque, 8012-95-1, JPN) for 44 h of culture. Then, COCs were treated with 0.1% hyaluronidase to remove the cumulus cells. Mature oocytes that released the first polar body in each treatment were calculated.

### Parthenogenesis of Oocyte

For parthenogenesis activation, mature oocytes were treated with A23187 (Calcium ionophore, Sigma, C-7522) for 5 min, 6-DAMP (6-dimethylamino purine, Sigma, D-2629) for 4 h, and PZM-3 (Porcine zygote medium-3) for 7 days. IVM tests were performed x 4 replicates for statistical analysis.

### Statistical Analysis

The maturation rate of oocyte and the blastocyst rate in different stages were analyzed by Duncan's new multiple range test using Statistical Analysis System, SAS, 9.4.*p* < 0.05 indicated significant difference.

## Results

**Figure 1:**
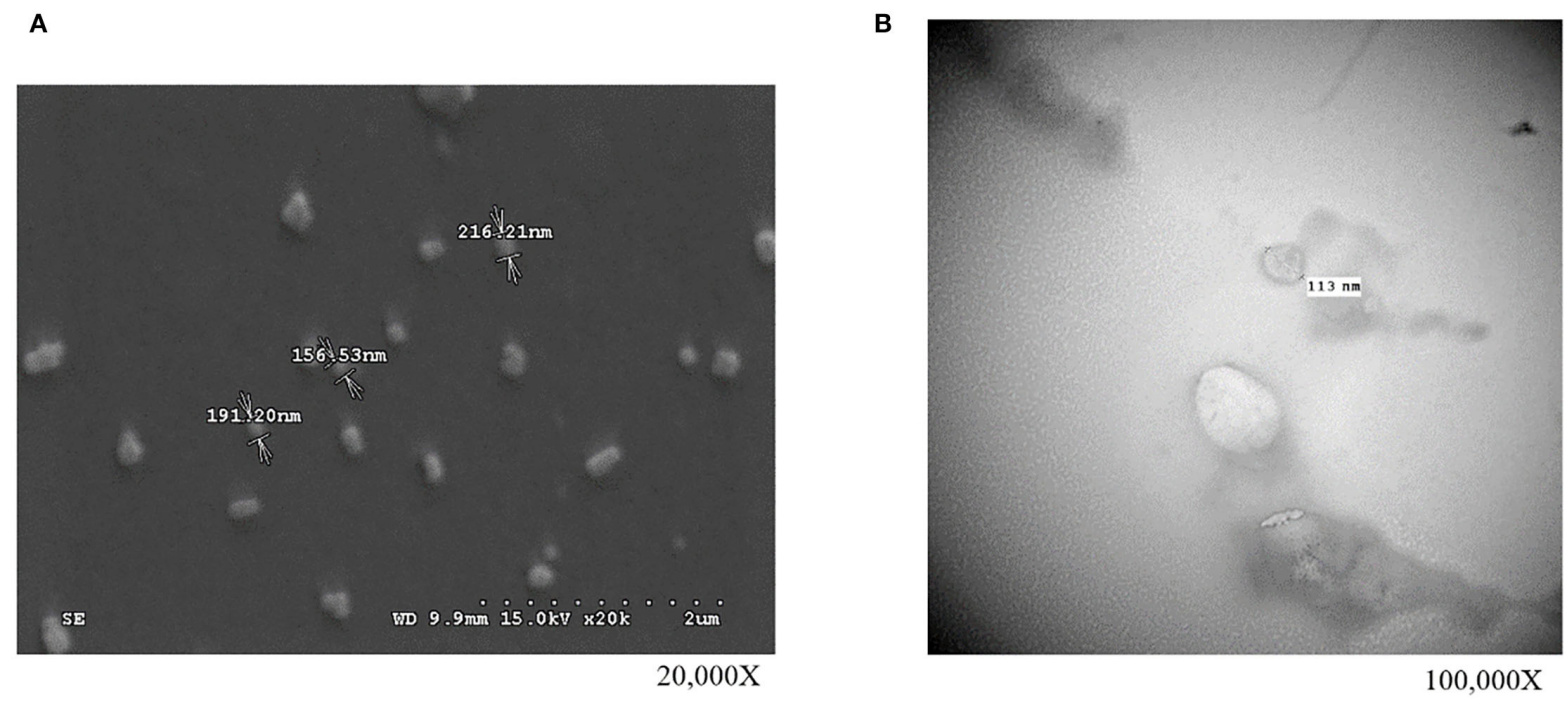
The observations of isolated EVs (collected from PFTSCs cultured medium) by high-resolution microscopies. Morphology and the diameter of EVs **(A)** under the scanning electron microscope (SEM) and **(B)** under the transmission electron microscopy (TEM) (repeated thrice).

**Figure 2:**
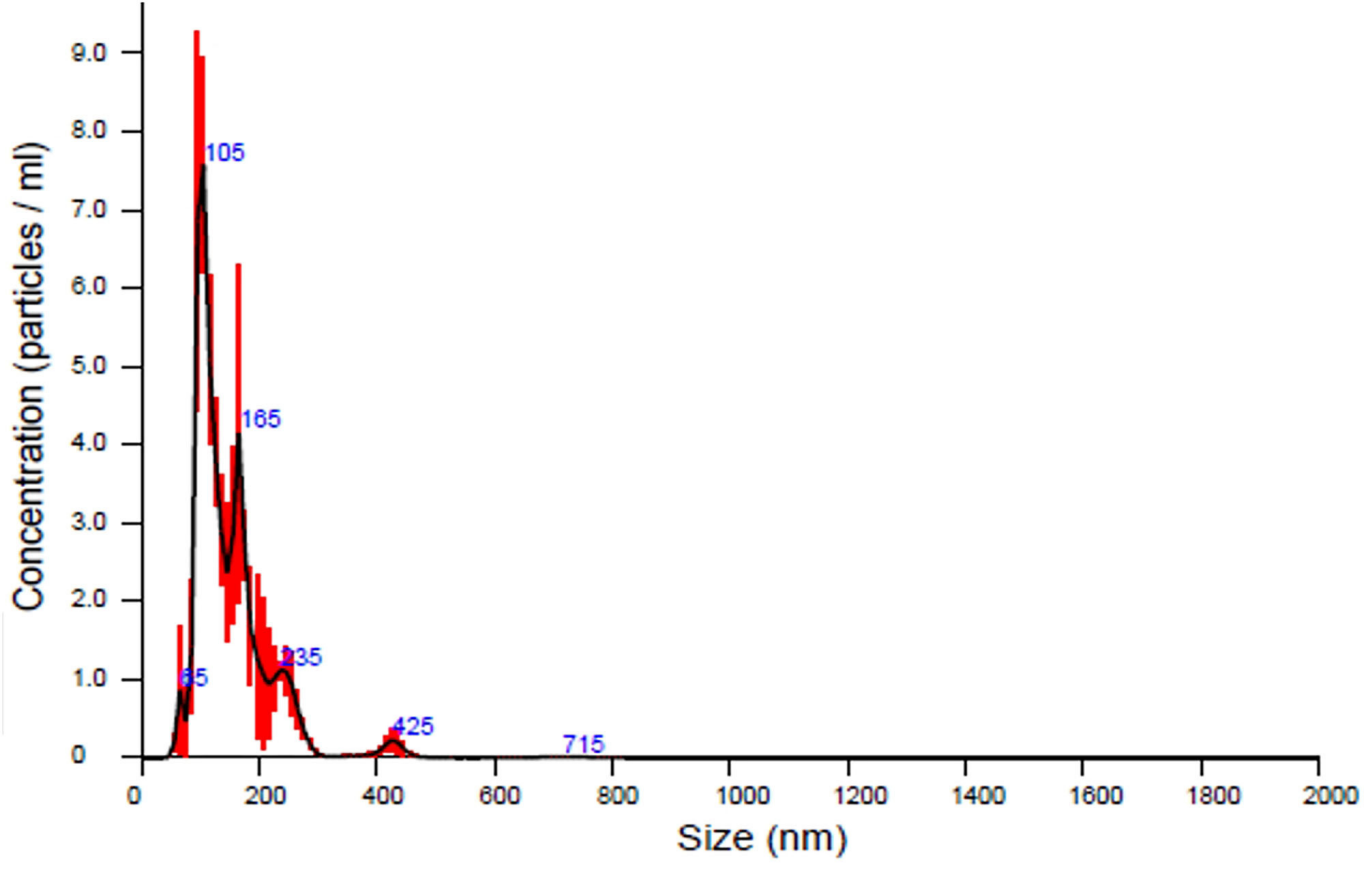
The analysis of the sizes of EVs secreted by PFTSCs which were collected and isolated from M199 medium (in each plate, and repeated thrice).

In our first experiment, the PFTSC-conditioned culture medium significantly elevated the maturation rates of oocytes (Table 1). That is, PFTSC might assist in the secretion to promote oocyte development. EVs from the culture medium were collected through the centrifugal approach (Figure 1). By SEM and TEM, the distribution of diameters of most EVs represented was between 100 and 200 nm and the average size was approximately 148 nm (Figure 2).

**Table 1:** Effects of IVM media supplemented with PFTSCs CM on the development of parthenogenetic activation porcine embryos.

**Treatment**	**Total No**.	**Maturation**	**Embryo**				**Cell No. of**
**group**	**of oocytes**	**rate (%)**	**stage of IVC**				**blastocysts**
			**2 cells (%)**	**4 cells (%)**	**8 cells (%)**	**Blastocysts (%)**	
IVM with M199	267	178 (66.7 ± 8.2)^b^	174 (97.7 ± 2.7)^a^	163 (91.4 ± 3.1)^a^	141 (79.2 ± 11.4)^a^	54 (30.1 ± 13.7)^a^	35.3 ± 2.2^a^
T1	260	200 (76.9 ± 2.1)^a^	188 (97.9 ± 1.1)^a^	176 (91.6 ± 2.4)^a^	159 (83.3 ± 1.6)^a^	51 (29.7 ± 4.6)^a^	36.6 ± 2.5^a^
T2	273	212 (77.7 ± 1.8)^a^	207 (98.2 ± 1.3)^a^	188 (89.3 ± 1.6)^a^	175 (83.2 ± 2.7)^a^	73 (35.1 ± 2.1)^a^	35.2 ± 1.6^a^

*T1, IVM with PFTSCs treated M199 medium (condition medium is kept at 4°C for at least 1 hour)*.

*T2, IVM with PFTSCs treated M199 medium (condition medium is kept at−80°C for at least 1 hour)*.

a, b *represents the significant difference (p < 0.05)*.

**Figure 3:**
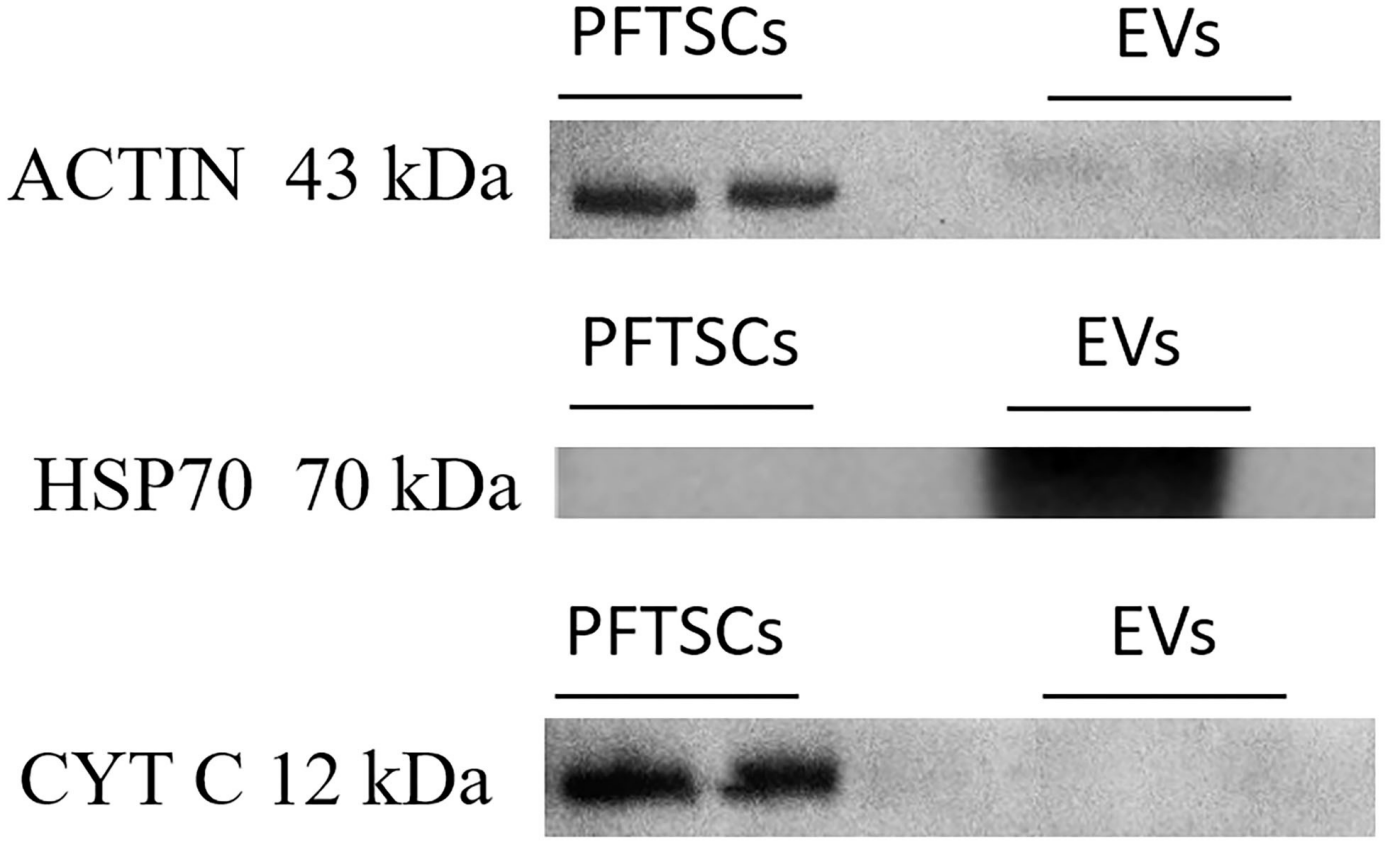
The characterization and differences between PFTSCs and collected EVs. The experiment was demonstrated by Western blotting (repeated thrice).

To validate the purity of EVs, three well-known markers (ACTIN, HSP70, and CYT C) for cells and EVs were examined by immunoblotting. All ACTIN expressed in cells and EVs was the most common housekeeping protein and cytoskeleton. HSP expressed only in normal proteins of EVs and was represented as negative control. CYT C expressed only in cells during apoptosis and was regarded as positive control (Figure 3). As the result, EVs collected by precipitation were not contaminated with cells. Identification of the existing miRNAs of EVs was conducted in the following analysis.

Of 457 porcine miRNAs in the current database, 267 miRNAs had been confirmed in the EVs from PFTSC. The most abundant 14 miRNAs were presented in Table 2.

**Table 2:** The list of 14 miRNAs with highest expressions among the 267 miRNAs found in isolated EVs.

**Name**	**Alias**	**Name**	**Alias**
ssc-miR-148a-3p	MIMAT0002124	ssc-let-7f-5p	MIMAT0002152
ssc-miR-10b	MIMAT0013885	ssc-miR-423-5p	MIMAT0013880
ssc-miR-99a-5p	MIMAT0013896	ssc-miR-143-3p	MIMAT0013879
ssc-miR-151-3p	MIMAT0013883	ssc-miR-320	MIMAT0013878
ssc-miR-378	MIMAT0013868	ssc-miR-27b-3p	MIMAT0013890
ssc-miR-140-3p	MIMAT0006786	ssc-miR-6529	MIMAT0041611
ssc-miR-152	MIMAT0013887	ssc-miR-22-3p	MIMAT0015710

*There are currently 457 miRNAs in the database miRNAs of pig. Among them, 267 miRNAs of EVs secreted by PFTSCs were analyzed, and the table showed the 14 types with the highest appearances of 267 types*.

**Figure 4:**
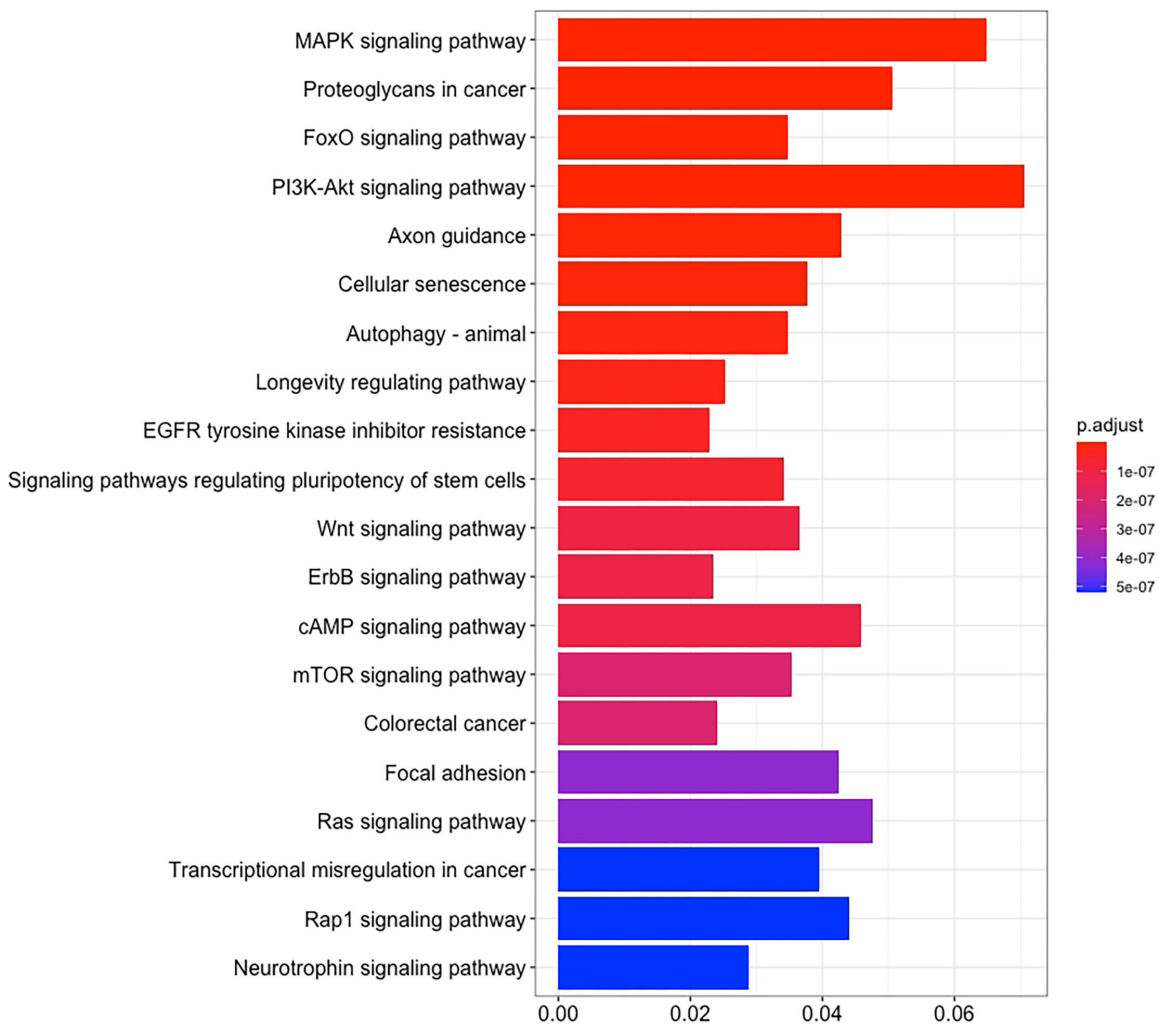
The enrichment maps of GO were analyzed from 14 miRNAs.

**Figure 5:**
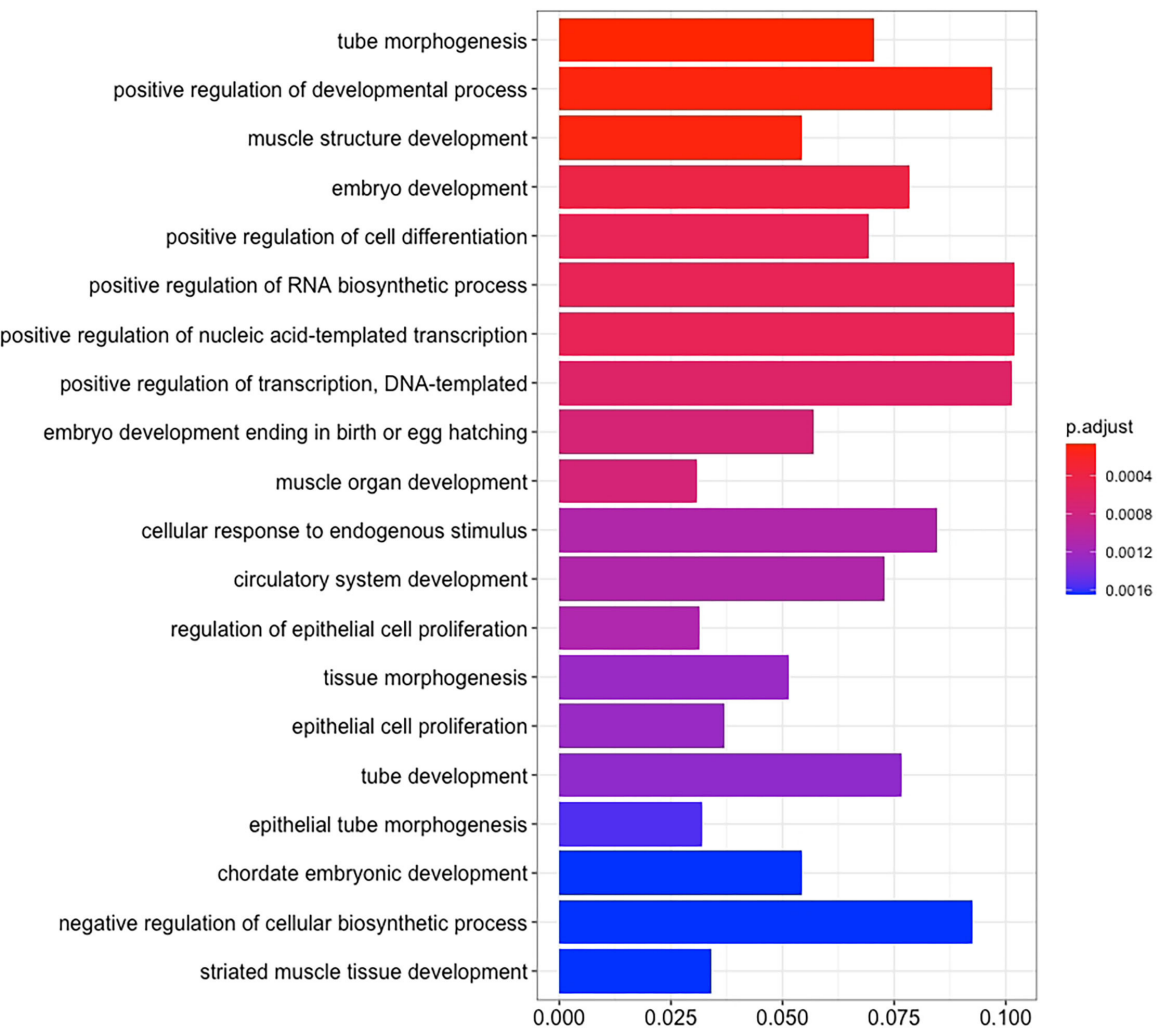
The enrichment maps of KEGG pathways were analyzed from 14 miRNAs.

There are 14 miRNAs were identified by analysis of GO and KEGG pathway which related to the oocyte development and meiosis (Figures 4, 5, Supplementary Table 1). In GO pathway analysis, this series of miRNAs was highly associated with oocyte development. Active modulation of embryo growth and cell differentiation, positive modulation of RNA synthesis and nucleic acid template transcription, promotion of DNA transcription, termination of embryo growth at birth or incubation, cellular response to endogenous stimulation, and negative modulation of cellular synthesis were observed. On the contrary, in KEGG pathway analysis, this series of miRNAs might combine with the genes related with MAPK signaling pathway, which is common in meiosis. The important genes in the meiosis of oocytes and the 14 abundant of miRNAs were compared to realize the regulation of miRNA on the meiosis related genes (Table 3). Cpeb1, an upstream gene in the MAPK pathway, and five related miRNAs, such as miR-152-3p, miR-148a-3p, miR-320a-3p, let-7f-5p, and miR-22-3p, served as the main subjects to analyze the developmental ability of embryo in parthenogenesis during the 44 h of *in vitro* maturation with the addition of miRNA at different concentration (Supplementary Table 2).

**Table 3:** Sequence selected of miRNAs.

**miRNAs**	**Sequence**
miR-152-3p	UCAGUGCAUGACAGAACUUGG
miR-148a-3p	UCAGUGCACUACAGAACUUUGU
miR-320a-3p	AAAAGCUGGGUUGAGAGGGCGA
let-7f-5p	UGAGGUAGUAGAUUGUAUAGUU
miR-22-3p	AAGCUGCCAGUUGAAGAACUGU

During the *in vitro* maturation, 2, 20, and 50 nM of five miRNAs were added. The maturation rate with different concentrations of miR-152-3p (76.0 ± 2.6–83.2 ± 2.8%) showed no significant difference in comparison with the control group (77.0 ± 6.0%) (Table 4). The maturation rate with different concentration of miR-148a-3p (72.6 ± 3.8–74.6 ± 4.8%) showed no significant difference in comparison with the control group (70.2 ± 2.6%) (Table 5). Surprisingly, the maturation rate with different concentration of miR-320a-3p (82.2 ± 4.0–83.9 ± 5.8%) showed a significant difference (Table 6). Furthermore, the blastocyst rate with 50 nm of miR-320a-3p (52.8 ± 3.8%) showed a significant difference (Tables 7, 8). The maturation rate with different concentration of miR-22-3p (73.9 ± 3.3–77.5 ± 4.1%) and let-7f-5p (68.2 ± 0.5–77.7 ± 3.1%) showed no significant difference either. Consequently, miR-320a-3p was selected to demonstrate the association between miRNA and maturation rate during *in vitro* cultivation (Table 9). After adding 2, 20, or 50 nM of miR-320a-3p, no significant difference was observed in the cleavage rate. However, the blastocyst rate of all groups adding miR-320a-3p (29.3 ± 2.4–31.3 ± 0.4%) was significantly higher than that of the control group (20.7 ± 3.9%).

**Table 4:** Effects of IVM media supplemented with miR-152-3p on the development of parthenogenetic activation porcine embryos.

**Treatment**	**Total No**.	**Maturation**	**Embryo**				**Cell No. of**
**group**	**of oocytes**	**rate (%)**	**stage of IVC**				**blastocysts**
			**2 cells (%)**	**4 cells (%)**	**8 cells (%)**	**Blastocysts (%)**	
IVM with M199	137	105 (77.0 ± 6.0)^a^	101 (96.0 ± 1.1)^a^	93 (88.7 ± 1.7)^a^	76 (72.5 ± 3.3)^a^	32 (30.0 ± 4.2)^a^	39.7 ± 2.5^a^
2 nM	138	105 (76.0 ± 2.6)^a^	95 (90.6 ± 1.2)^a^	87 (83.2 ± 2.4)^a^	72 (69.0 ± 3.1)^a^	39 (37.4 ± 4.5)^a^	35.7 ± 2.1^a^
20 nM	136	113 (83.2 ± 2.8)^a^	101 (89.4 ± 2.2)^a^	93 (82.3 ± 1.2)^a^	77 (68.0 ± 3.0)^a^	32 (28.2 ± 4.6)^a^	34.7 ± 2.8^a^
50 nM	127	103 (81.3 ± 2.1)^a^	97 (94.2 ± 1.8)^a^	90 (87.6 ± 2.7)^a^	82 (79.8 ± 3.3)^a^	38 (37.2 ± 6.3)^a^	35.3 ± 2.3^a^

a *represents the significant difference (p < 0.05)*.

**Table 5:** Effects of IVM media supplemented with miR-148a-3p on the development of parthenogenetic activation porcine embryos.

**Treatment**	**Total No**.	**Maturation**	**Embryo**				**Cell No. of**
**group**	**of oocytes**	**rate (%)**	**stage of IVC**				**Blastocysts**
			**2 cells (%)**	**4 cells (%)**	**8 cells (%)**	**Blastocysts (%)**	
IVM with M199	186	130 (70.2 ± 2.6)^a^	127 (97.8 ± 1.4)^a^	115 (88.6 ± 2.0)^a^	92 (70.7 ± 2.8)^a^	45 (34.7 ± 5.0)^a^	35.6 ± 2.3^a^
2 nM	189	140 (74.6 ± 4.8)^a^	135 (96.7 ± 2.0)^a^	119 (85.5 ± 3.3)^a^	105 (75.7 ± 6.3)^a^	53 (38.5 ± 4.2)^a^	40.8 ± 2.5^a^
20 nM	186	134 (72.6 ± 3.8)^a^	126 (94.0 ± 3.5)^a^	115 (85.7 ± 6.1)^a^	101 (75.0 ± 10.2)^a^	56 (41.7 ± 7.8)^a^	37.5 ± 2.4^a^
50 nM	185	135 (73.5 ± 6.1)^a^	129 (95.6 ± 3.0)^a^	119 (88.2 ± 5.0)^a^	102 (76.0 ± 4.2)^a^	61 (46.4 ± 5.4) ^a^	41.8 ± 2.0^a^

a *represents the significant difference (p < 0.05)*.

**Table 6:** Effects of IVM media supplemented with miR-320a-3p on the development of parthenogenetic activation porcine embryos.

**Treatment**	**Total No**.	**Maturation**	**Embryo**				**Cell No. of**
**group**	**of oocytes**	**rate (%)**	**stage of IVC**				**blastocysts**
			**2 cells (%)**	**4 cells (%)**	**8 cells (%)**	**Blastocysts (%)**	
IVM with M199	159	111 (69.9 ± 1.9)^b^	104 (93.7 ± 0.9)^a^	94 (84.6 ± 2.5)^a^	79 (71 ± 4.4)^a^	35 (31.4 ± 6.1)^b^	37.9 ± 2.9^a^
2 nM	161	135 (83.9 ± 5.8)^a^	131 (97.0 ± 0.7)^a^	117 (87 ± 2.5)^a^	101 (75 ± 4.2)^a^	53 (39.2 ± 0.4)^ab^	39.1 ± 2.2^a^
20 nM	160	134 (83.8 ± 1.0)^a^	129 (96.3 ± 2.7)^a^	120 (89.6 ± 2.9)^a^	110 (82.1 ± 2.1)^a^	56 (39.2 ± 0.4)^ab^	38.0 ± 2.0^a^
50 nM	170	140 (82.2 ± 4.0)^a^	137 (98.0 ± 1.2)^a^	129 (92.5 ± 2.6)^a^	116 (83.3 ± 3.1)^a^	74 (52.8 ± 3.8)^a^	41.7 ± 2.3^a^

a, b *represents the significant difference (p < 0.05)*.

**Table 7:** Effects of IVM media supplemented with miR-22-3p on the development of parthenogenetic activation porcine embryos.

**Treatment**	**Total No**.	**Maturation**	**Embryo**				**Cell No. of**
**group**	**of oocytes**	**rate (%)**	**stage of IVC**				**blastocysts**
			**2 cells (%)**	**4 cells (%)**	**8 cells (%)**	**Blastocysts (%)**	
IVM with M199	147	114 (77.5 ± 4.1)^a^	103 (90.1 ± 2.6)^a^	91 (79.7 ± 2)^a^	76 (66.3 ± 3.9)^a^	22 (19.0 ± 2.6)^a^	38.0 ± 2.8^a^
2 nM	141	104 (73.9 ± 3.3)^a^	100 (96.1 ± 1.1)^a^	83 (79.5 ± 6.6)^a^	76 (72.7 ± 6.6)^a^	27 (25.7 ± 6.7)^a^	33.5 ± 2.4^a^
20 nM	141	108 (76.9 ± 4.7)^a^	101 (93.4 ± 2.6)^a^	88 (81.3 ± 1.6)^a^	75 (69.3 ± 2.3)^a^	35 (32.1 ± 5.2)^a^	38.2 ± 2.2^a^
50 nM	141	135 (79.5 ± 2.8)^a^	105 (93.7 ± 2.4)^a^	99 (88.3 ± 4)^a^	85 (75.8 ± 2.8)^a^	35 (31.2 ± 3.5)^a^	41.5 ± 3.0^a^

a *represents the significant difference (p < 0.05)*.

**Table 8:** Effects of IVM media supplemented with let-7f-5p on the development of parthenogenetic activation porcine embryos.

**Treatment**	**Total No**.	**Maturation**	**Embryo**				**Cell No. of**
**group**	**of oocytes**	**rate (%)**	**stage of IVC**				**blastocysts**
			**2 cells (%)**	**4 cells (%)**	**8 cells (%)**	**Blastocysts (%)**	
IVM with M199	182	130 (72.7 ± 4.9)^a^	117 (90.5 ± 3.1)^a^	104 (80.3 ± 3.2)^a^	95 (73.4 ± 4.2)^a^	44 (33.6 ± 3.2)^a^	39.8 ± 2.5^a^
2 nM	183	125 (68.2 ± 0.5)^a^	115 (92 ± 1.5)^a^	109 (86.7 ± 2.9)^a^	94 (76.4 ± 5.1)^a^	33 (26.5 ± 2.8)^a^	43.1 ± 3.2^a^
20 nM	185	142 (77.7 ± 3.1)^a^	139 (98.3 ± 1.7)^a^	129 (91.2 ± 1.6)^a^	123 (87.1 ± 2.3)^a^	38 (27.5 ± 6.5)^a^	36.4 ± 2.1^a^
50 nM	183	128 (70.3 ± 1.3)^a^	121 (95.1 ± 2.3)^a^	107 (84.7 ± 4.9)^a^	95 (75.6 ± 5.9)^a^	38 (29.1 ± 3.8)^a^	37.5 ± 2.1^a^

a *represents the significant difference (p < 0.05)*.

**Table 9:** Effects of IVC media supplemented with miR-320a-3p on the development of parthenogenetic activation porcine embryos.

**Treatment**	**Total No**.	**Embryo**					**Cell No. of**
**group**	**of matured oocytes**	**stage after IVC**					**Blastocysts**
		**2 cells (%)**	**4 cells (%)**	**8 cells (%)**	**Morula (%)**	**Blastocysts (%)**	
IVM with M199	168	158 (94.6 ± 1.8)^a^	135 (81.6 ± 3.8)^a^	99 (59.8 ± 2.8)^a^	74 (44.8 ± 3.0)	35 (20.7 ± 3.9)^b^	46.6 ± 2.5^a^
2 nM	163	153 (93.7 ± 0.8)^a^	138 (83.5 ± 3.8)^a^	119 (71.3 ± 5.5)^a^	92 (55.5 ± 3.1)	51 (31.3 ± 0.4)^a^	44.3 ± 2.3^a^
20 nM	169	164 (97.2 ± 1.7)^a^	139 (81.9 ± 1.7)^a^	117 (69.3 ± 1.0)^a^	91 (54.0 ± 0.6)	49 (29.3 ± 2.4)^a^	43.6 ± 2.7^a^
50 nM	162	152 (93.6 ± 1.5)^a^	133 (81.2 ± 2.8)^a^	105 (63.4 ± 4.7)^a^	84 (49.9 ± 6.1)	50 (30.3 ± 1.7)^a^	44.7 ± 2.7^a^

a, b * represents the significant difference (p < 0.05)*.

## Discussion

Based on previous study, the diameter of EVs from bovine, murine, and human were 30–250 nm, 25–100 nm, and 50–250 nm, respectively (8, 15, 16). Consequently, extracellular follicles from fallopian tubes of different animals had similar diameters. Previous studies suggested that EVs could affect target cells. For example, EVs from human corneal stem cell repair the corneal lesion (17). The addition of EVs from bovine fallopian tube epithelium into *in vitro* culture (IVC) medium improves the quality of embryos. MiRNA of EVs from bovine follicular fluid modulates the maturation of follicles (18). Therefore, next experiment aimed to analyze the miRNAs of EVs from PFTSC.

Cpeb1, an upstream gene in the MAPK pathway, adjust the growth of oocyte and follicle and manifest the stability and translation of mRNA of oocytes (19–21). Several miRNAs, such as miR-152-3p, miR-148a-3p, miR-320a-3p, let-7f-5p, and miR-22-3p are predicted to targeting Cpeb1. Additionally, cell cycle regulatory genes Ccnb1, Ccnb2, and Ccnb3 have been shown to regulate by miR-27b-3p, miR-10b-5p, miR-148a-3p, miR-152-3p, and miR-22-3p (22–25). Taf4 was the gene to activate the translation of oocyte maturation which was modulated by miR-152-3pand miR-148a-3p (26, 27). CDh1 the gene related to the modification of mRNA translation during the maturation of oocyte was regulated by miR-148a-3p (28, 29). phosphatase and tensin homolog (Pten) was suggested to reduce the oxidative stress of oocytes which was affected by miR-152-3p, miR-148a-3p, miR-320a-3p, and miR-22-3p (30–32). The above genes were highly associated with the meiosis of oocytes.

Certain medicine may up-regulate the expression of let-7f-5p in tumor cells. For example, the expressions of TP53, TP53INP1, and TP53INP2 were increased after the cultivation with let-7f-5p for 48 hours, which promoted the progress of apoptosis. Therefore, let-7f-5p was able to assist chemotherapy to treat cancer (33). The expression of miR-22-3p inactivated AKT3 to inhibit the proliferation of cancer cell (34). Besides, miR-22-3p might increase Pten and inhibit apoptosis (35). Ganglion cells exposed to 200 μM miR-22 could inhibit the expression of Cpeb (36).

These miRNAs could affect several genes, such as Cpeb1, Ccnb, and Pten (37). Phosphorylated Cpeb1 activated MAPK pathway to promote the maturation of oocytes (19, 38). The group of genes associated with oocyte maturation was highly assumed to be regulated by miR-320a-3p. miR-320a-3p not only matures the nucleus of oocytes, but also induces the development of cytoplasm to decrease the injury from the discharge of polar body. Then, the potential of oocyte growth and blastocyst rate would be elevated. Thus, no significant difference in other miRNAs groups might be derived from insufficient concentration. The mixture of multiple miRNAs might demonstrate effectiveness.

The aims of this study were to investigate the association of EVs from PFTSC and the oocytes. Furthermore, the miRNAs of EVs were analyzed to identify the key factor of the modification of oocyte growth. The expression of ACTIN, CYT C, and HSP70 was utilized to determine the contamination of cells. The average diameter of EV collected from CM was 148 nm, ranging from 100 to 200 nm. According to the current database, 267 of 457 miRNAs were identified in this study. The most abundant 14 miRNAs were highly associated with oocyte development. During the *in vitro* maturation of miR-152-3p, miR-148a-3p, miR-320a-3p,let-7f-5p, and miR-22-3p, the maturation rate of miR-320a-3p group was significantly higher than that of control group. Additionally, the blastocyst rate of parthenogenesis in the group with 50 nM miR-320a-3p was significantly higher. Other miRNAs showed no significant difference in maturation, oocyte, blastocyst rate, and cell numbers of blastocysts. In *in vitro* cultivation, miR-320a-3p group showed a significant difference in blastocyst rate but not in cleavage rate and cell numbers of blastocysts.

## Conclusion

The conditioned medium of PFTSC was able to assist in the development of oocytes. Also, miR-320a-3p of EVs from the medium could elevate the oocyte maturation and blastocyst rate. That is, fallopian tube secreted essential miRNAs to modify the oocyte and embryo development. In order to increase the oocyte quality and decrease the loss of embryo production, the environment of *in vitro* fertilization should be improved. This technique not only shares the potential to elevate the successful rate of artificial fertilization, but also preserves the specific husbandry animal population in the future.

## Data Availability Statement

The original contributions presented in the study are included in the article/Supplementary Materials, further inquiries can be directed to the corresponding authors.

## Author Contributions

T-YF, S-HW, SS, and S-YP designed the study. SS and S-YP coordinated the study and critically reviewed the manuscript. T-YF, S-HW, P-CS, and S-CC performed the experiments. T-YF, Y-HL, and C-JC analyzed the data. S-HW, Y-HY, K-TY, and C-WH interpreted the data. T-YF and S-HW drafted the manuscript. T-YL revised the manuscript. All authors were involved in the amendment and approval of final manuscript.

## Funding

This work was supported to SS who is the PI by Chang Gung Memorial Hospital grant CPRPG1K0012, CMRPG1K0201 and Ministry of Science and Technology Taiwan grant MOST 108-2314-B-182A-085. This work was also supported to S-YP who is the PI from National Pingtung University of Science and Technology and by Ministry of Science and Technology Taiwan grant MOST 108-2313-B-020-007-MY3.

## Conflict of Interest

The authors declare that the research was conducted in the absence of any commercial or financial relationships that could be construed as a potential conflict of interest.

## Publisher's Note

All claims expressed in this article are solely those of the authors and do not necessarily represent those of their affiliated organizations, or those of the publisher, the editors and the reviewers. Any product that may be evaluated in this article, or claim that may be made by its manufacturer, is not guaranteed or endorsed by the publisher.

## Supplementary Material

Supplementary Table 1 The list of 267 miRNAs identified by next generation sequencing.

Supplementary Table 2 The list of targeted genes affecting MAPK pathway.
